# Ozone in Medicine. The Low-Dose Ozone Concept and Its Basic Biochemical Mechanisms of Action in Chronic Inflammatory Diseases

**DOI:** 10.3390/ijms22157890

**Published:** 2021-07-23

**Authors:** Renate Viebahn-Haensler, Olga Sonia León Fernández

**Affiliations:** 1 Medical Society for the Use of Ozone in Prevention and Therapy, Iffezheim, D-76473 Baden-Baden, Germany; 2 Pharmacy and Food Institute, University of Havana, Coronela, Lisa, Havana 10 400, Cuba

**Keywords:** ozone therapy, bioregulation, redox balance, chronic inflammation, biological medicine, complementary medicine

## Abstract

Low-dose ozone acts as a bioregulator in chronic inflammatory diseases, biochemically characterized by high oxidative stress and a blocked regulation. During systemic applications, “Ozone peroxides” are able to replace H_2_O_2_ in its specific function of regulation, restore redox signaling, and improve the antioxidant capacity. Two different mechanisms have to be understood. Firstly, there is the direct mechanism, used in topical treatments, mostly via radical reactions. In systemic treatments, the indirect, ionic mechanism is to be discussed: “ozone peroxide” will be directly reduced by the glutathione system, informing the nuclear factors to start the regulation. The GSH/GSSG balance outlines the ozone dose and concentration limiting factor. Antioxidants are regulated, and in the case of inflammatory diseases up-regulated; cytokines are modulated, here downregulated. Rheumatoid arthritis RA as a model for chronic inflammation: RA, in preclinical and clinical trials, reflects the pharmacology of ozone in a typical manner: SOD (superoxide dismutase), CAT (catalase) and finally GSH (reduced glutathione) increase, followed by a significant reduction of oxidative stress. Inflammatory cytokines are downregulated. Accordingly, the clinical status improves. The pharmacological background investigated in a remarkable number of cell experiments, preclinical and clinical trials is well documented and published in internationally peer reviewed journals. This should encourage clinicians to set up clinical trials with chronic inflammatory diseases integrating medical ozone as a complement.

## 1. Introduction

Basic reaction mechanisms of ozone originate from research and development laboratories in the water treatment environment: reaction constants and mechanisms for a variety of inorganic and organic substrates, bactericidal, fungicidal, or antiviral activity. Incorrectly, they are mostly transferred directly to medical applications and diseases. Here, we will show that medical ozone at low concentration and dosage in the form of “ozone peroxide” replaces the biological redox and immune regulation of hydrogen peroxide. This is of special interest in chronic inflammatory diseases with high oxidative stress, antioxidant deficiency, and immune imbalance, a condition in which biological regulation is largely disrupted.

The specific reaction of ozone with isolated double bonds of mono-unsaturated fatty acids to short-chain “ozone peroxides” (not with polyunsaturated fatty acids) induces the further reaction event in interaction with glutathione. To demonstrate this, the authors have evaluated their own essential investigations and publications and consulted the relevant publications of other authors.

### 1.1. Ozone as Bioregulator

The biochemistry of life is a kaleidoscope of dynamic processes, dynamically interacting equilibria in an almost confusing network. If individual cycles, partial balances, are disturbed, this biological network is still able to compensate for disturbances, repair defects, and keep the overall system “viable” for a long time.

Long-lasting disturbances, chronic stress, a multitude of different disturbance factors at various points of our biological network leave lesions within single or multiple dynamic, biochemical equilibria that can no longer be compensated and block the biological repair mechanisms. This mostly leads to chronic diseases.

Reactivating biological processes and, if possible, restarting the repair mechanisms is the target of Biological Medicine. In diseases with chronic oxidative stress, medical ozone has a special significance:

At low concentrations and dosages, it acts as a bioregulator, while the regulation is blocked when high concentrations are used.

In a biological system that can still be regulated, this role is incumbent on hydrogen peroxide H_2_O_2_, which is one of the most important oxidative bioregulators at biological concentrations [1,2]. When and if the H_2_O_2_ concentration is too high, regulation is blocked and degenerative processes begin, as in the case of chronic inflammatory diseases.

**Figure 1:**
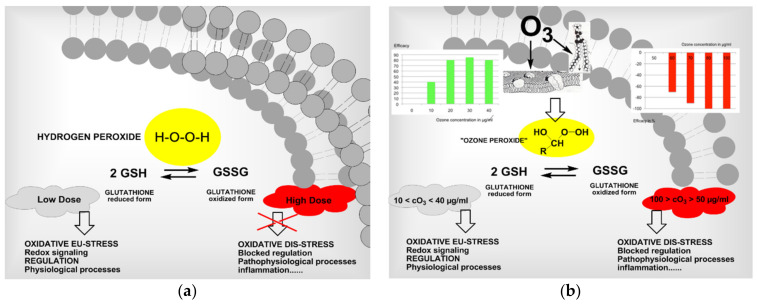
(**a**) Hydrogen peroxide as key redox regulator in the biological system modified acc. to [1,2]. (**b**). Low-dose ozone as bioregulator.

This key position of hydrogen peroxide is then likely to be assumed by the “ozone peroxide” formed from ozone, as seen in Figure 1a,b. Here, we will demonstrate this on a molecular and biochemical basis, supported by preclinical and clinical data using a prime example of a chronic inflammatory disease, rheumatoid arthritis.

### 1.2. Indications and Applications of Systemically Administered Ozone

Over the last three decades, the pharmacology and molecular mechanisms of ozone in medical use have been widely investigated and well confirmed. In the 1980s and 1990s its influence on the RBC (red blood cell) metabolism with an improvement of oxygen release as final result [3,4,5], while the impact of ozone on immunocompetent cells was the main subject of Bocci’s ozone research in the 1990s, e.g., [6,7], expanding the knowledge and understanding of immunomodulation.

The first paper on ozone as a bioregulator of the redox balance in a biological system was published by León and her group in 1998 followed by an intensive research program all over the world until today [8].

**Figure 2:**
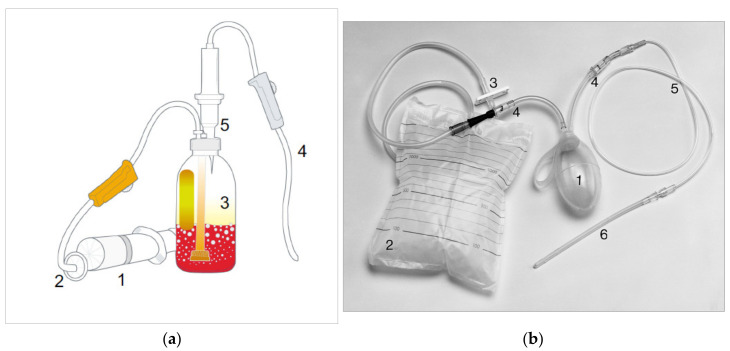
(**a**) [9] Major autohemotherapy. Standardized system acc. to the Medical Device Directives MDD 93/42EC. 1. Ozone syringe (PP, siliconized). 2. Bacterial filter 0,2 µ (germstop). 3. Vacuum bottle (glass) with micro bubble system (PP) 4. Transfusion set (PE). 5. Latex-free plug. (**b**) [9] Set for rectal insufflation, standardized system acc. to the Medical Device Directives 93/42 EC. 1. Dosage pump (silicone). 2. storage bag. 3. Clamp. 4. one-way valve. 5. connection line (PE). 6. catheter (PE).

On this basis and with standardized forms of ozone treatments, such as major auto-hemotherapy (MAH) and rectal insufflation (RI) as low-risk systemic ozone applications (see Figure 2a,b), the indications of medical ozone have been established, mainly chronic inflammations or diseases associated with chronic inflammatory conditions [9]. Following the low-dose ozone concept, medical ozone can be successfully integrated into a complementary medical system in angiopathia, rheumatoid arthritis, or chronic intestinal diseases as examples, see Table 1. They all have one phenomenon in common: high oxidative stress, measured as ROS (reactive oxygen species) and a suppressed antioxidant capacity as well as an immune disbalance

### 1.3. Procedures

MAH: 50–100 mL venous blood are withdrawn from the patient into the vacuum bottle and, as a rule, 50–100 mL ozone/oxygen mixture is bubbled through using sodium citrate as anticoagulant, to be immediately re-infused in a closed system under strict aseptic conditions; see Figure 2a. As ozone reacts in less than a second with blood components, such as unsaturated fatty acids in the cell membrane, not even one single ozone molecule enters the vascular system. The oxygen component of the O_2_/O_3_ mixture serves as carrier substance only and, with its poor solubility in blood, passes the liquid, remains in the bottle, and is not infused into the patient.

### 1.4. Ozone Concentration and Dosage in MAH

As summarized and discussed in the guidelines for the low-dose ozone concept [9], the ozone concentration range for systemic applications in the form of MAH should not exceed 10–40 µg/mL. Beyond 50 µg/mL, a toxic effect of ozone must be taken into account, and 80 µg ozone per mL of blood must never be used.

The ozone dosage per treatment will cover a range between 500 and 4000 µg. See Table 2 where we discuss:

- µg ozone per mL ozone/oxygen mixture which is delivered by the ozone generator,
- µg ozone per mL blood, or
- the total quantity of ozone in µg per total quantity of blood, or the total quantity of ozone in µg per treatment.

In medicine we normally use the unit 1 µg/mL, identical to 1 mg/L = 1 g/m^3^ used in waste water and drinking water purification.

Method. Rectal ozone insufflation can be given as an intestinal enema via a 30, 50, or 200 mL enema syringe or a special rectal set in order to administer 150 or 300 mL consisting of: an ozone supply container with lock valve, dosing pump with non-return valves, connecting tube with luer lock or 50 mL silicone-coated disposable syringe, and rectal catheter; see Figure 2b.

### 1.5. Dosage

- Systemic: 10–25 µg ozone/mL oxygen gas mixture, volume 150–300 mL; for children: 10–20 µg/mL, volume 10–30 mL; RI is the preferred systemic form of ozone application for children.
- Local: in ulcerous colitis, high O_3_/O_2_ concentrations (70–80–100 µg/mL) and small volumes (50 mL) are applied. On cessation of hemorrhage, this is reduced to 20–30 µg/mL, followed by systemic efficacy: 10–20 µg/mL, 150–300 mL volume.
- Rectal ozone application is simple, low-cost, and practically free of adverse reactions when dosages are adhered to exactly.
- As an adjuvant therapy in proctitis and proctocolitis, rectal insufflation is scientifically founded and to be recommended. Rectal O_3_ insufflation is increasingly being used in pediatrics, sports medicine, geriatrics, and as a complementary method in oncology.

## 2. Results

### 2.1. Mechanisms of Action

#### 2.1.1. The Reactivity and the Effects of Ozone

**Figure 3:**
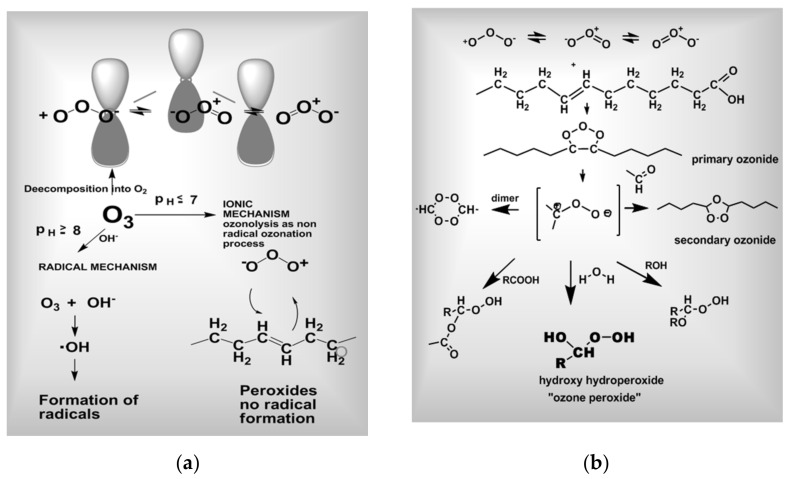
(**a**) Molecular structure. Radical and ionic reaction mechanisms of ozone modified after [9]. (**b**) Ozone reaction with isolated double bonds, i.e., ozonolysis according to Criegee 1953, 1975 [11,12]. The hydroxy hydroperoxides, here, “ozone peroxides” are understood as the pharmacologically active substances.

According to its reaction partners, ozone follows different reaction mechanisms. In the presence of organic reactants ozone has a markedly selective set of reactions [10]. In principle, radical and ionic reaction mechanisms are possible; see Figure 3a,b.

Decomposition of ozone into molecular oxygen with a system-dependent half-life (2 O_3_ → 3 O_2_) in the gaseous state occurs, e.g., t_1/2_ = 55 min, a disposable 50 mL syringe (polypropylene and siliconized piston) or also in aqueous medium, though with a considerably prolonged half-life, e.g., t_1/2_ = 10 h in double distilled water with a conductivity of 0.05 µS at 20 °C/68 °F.

Due its high reactivity with isolated double bonds in unsaturated fatty acids (UFA), ozone reacts under physiological conditions (MAH or RI) in less than a second in a highly specific manner according to classical ozonolysis, namely the “Criegee mechanism”, an ionic, non-radical reaction [11,12]. Starting with the 1.3 dipolar electrophilic addition to isolated C=C double bonds short-chain hydroxy-hydroperoxides, here designated as “ozone peroxides”, are formed as intermediates at pH ≤ 7.4 as shown in Figure 3a,b.

It is very likely the “ozone peroxide” takes over the role of a second messenger substance inducing most of the pharmacological effects via glutathione as the high effective and central redox system of each cell.

Contrary to the small and transient oxidative stress obtained with low ozone concentrations and the small amount of “ozone peroxides” with their terminal peroxide group, the permanent oxidative stress in the organism is produced by oxygen and/or OH radicals with e.g., polyunsaturated fatty acids (PUFA) forming long chain peroxides with a central peroxide group. These relatively stable peroxides with the tendency to set off radical chain reactions are long living compounds and are taken as reference substances (measured as total hydroperoxides TH) for an oxidative stress situation in chronic inflammatory diseases, the main indication of ozone therapy.

Ozone itself does not preferably react with PUFAs, especially with conjugated double bonds due to their quantum mechanical stability. That means it does not contribute to oxidative stress if proper concentrations and dosages are used as experienced in the low-dose ozone concept.

#### 2.1.2. Direct Effects: Germicidal and Virus-Inactivating Effects in Topical Ozone Applications

Using ozone in medicine, we strictly have to distinguish between its direct and indirect effects.

The bactericidal and fungicidal as well as virus inactivating effects, well known from water purification and drinking water treatment, are used in heavily infected wounds such as burns, diabetic foot, and ulcus cruris, and becoming increasingly important in lesions infected with germs resistant to most of the antibiotics such as MRSA (methicilline-resistant Staphylococcus aureus).

**Figure 4:**
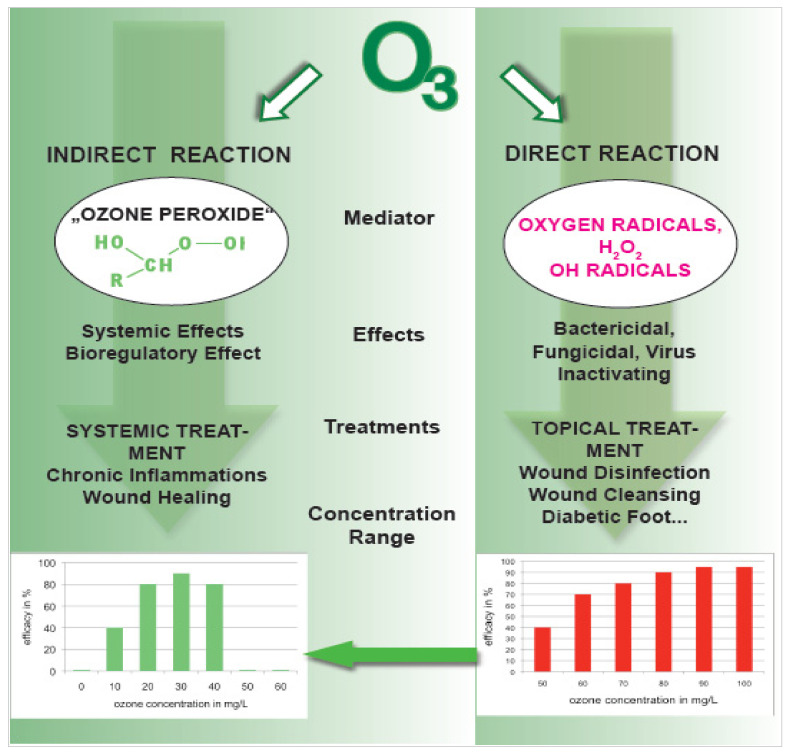
Direct and indirect ozone reactions in topical and systemic treatment within the corresponding concentration ranges.

Here, we make use of the high concentration range by topical treatment in the form of “gas-bagging“ or the low-pressure treatment method according to Werkmeister [13] for disinfecting and wound cleansing. The formation of different oxygen and finally OH-radicals is the dominating ozone mechanism of action with a direct impact on bacteria, microorganisms, and viruses. For wound healing, a low concentration range is necessary to make use of with the indirect ozone reaction; see Figure 4.

#### 2.1.3. Indirect Effects in Systemic Treatments: Signal Transduction and Bioregulation

**Figure 5:**
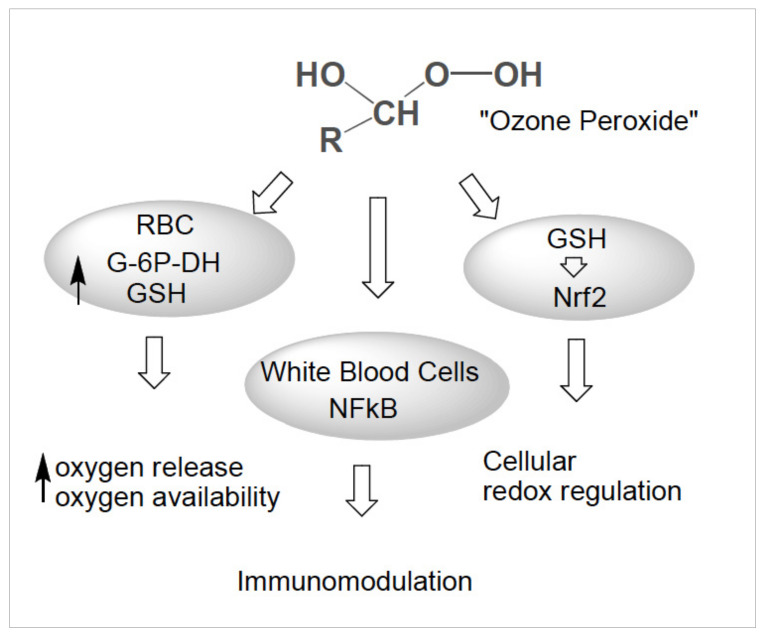
The pharmacological effects of medical ozone via ozone-produced peroxides. The three effects: 1. improved oxygen release by the red blood cells (RBC) through activation of RBC metabolism. 2. Immunomodulation through activation of the white blood cells (WBC) and signal transduction via nuclear factors. 3. Regulation of cellular antioxidants via Nrf2 signalling [9].

At low doses, systemically applied ozone in the form of major autohemotherapy (MAH) and rectal insufflation (RI) acts as a bioregulator, as schematically shown in Figure 5: as a reaction product from O_3_ and unsaturated fatty acids (isolated double bonds), “ozone peroxides“ induce a signal transduction by the oxidation of glutathione (or cysteine residues), informing the corresponding nuclear factors, finally resulting in a regulation of the antioxidants via Nrf2 (transcription factor: nuclear factor erythroid 2-related factor 2), or an immunomodulation via NFkB (transcription factor: nuclear factor kB).

In addition to the indication-relevant clinical parameters, the course of treatment can be followed by monitoring specific antioxidants and characteristic cytokines.

#### 2.1.4. Dose-Response Relationship

**Figure 6:**
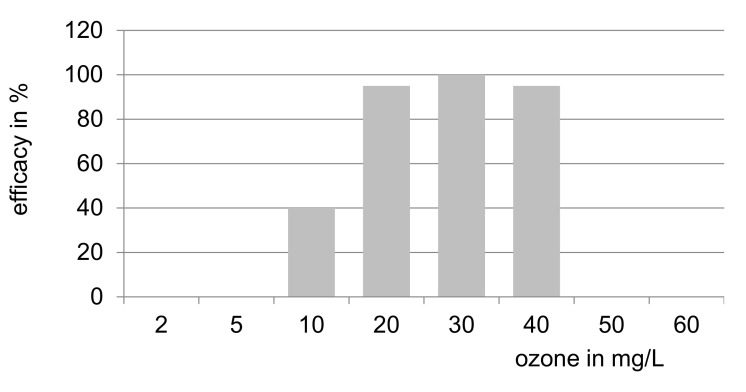
Dose response: efficacy–concentration relationship of systemically administered medical ozone as hormetic substance (schematically), modified after [9].

In its dose–response medical ozone follows the principle of hormesis: low concentrations (or doses) show a high efficacy, which decreases with increasing concentrations, finally reversing into an ineffective and even toxic effect. Figure 6 shows the efficacy/concentration relationship for the systemic application of ozone. Here, concentrations of 10–40 µg/mL represent those levels which are physiologically effective and recommended for systemic application.

Concentrations below 10 µg/mL have a very low or no impact, as antioxidants in the serum such as Vit. C will scavenge ozone completely. Systemically administered concentrations ≥ 60 µg/mL are toxic. The antibiotic effect of ozone in the concentration range of 60 to 100 µg/mL is completely restricted to the topical forms of application.

## 3. Discussion

### 3.1. “Ozone Peroxides” as Signal Molecules

#### 3.1.1. Oxidative Stress

High oxidative stress and an insufficient antioxidant system are a biochemical characteristic of chronic inflammatory diseases whereby biological regulation is overstrained and partly or completely inhibited. Here, one may ask whether systemic ozone application using “active oxygen” is really appropriate. Does this not increase H_2_O_2_, MDA (malondialdehyde), and TH (total hydroperoxides), producing a further oxidative stressor, finally destroying the antioxidant system already working beyond its capacity in these indications?

Total hydroperoxides, the end product of polyunsaturated fatty acids (PUFA) plus OH-radicals and/or oxygen (autoxidation as radical reaction), can be taken as a significant parameter for chronic oxidative stress. These long-chain peroxides with a central hydro peroxide group have a considerable half-life in the biological system, and initiate in their turn radical chain reactions.

#### 3.1.2. “Ozone Peroxides” as Second Messenger Molecules

Autoxidation is not to be confused with ozonolysis (Figure 3b): whereas ozone reacts with isolated double bonds in a highly selective manner, this is not or far less the case with polyunsaturated fatty acids, i.e., especially conjugated double bonds.

As a short-chain, non-radical peroxide with the -OOH group in the end position, the “ozone peroxide” R-CH(OH)-OOH belongs to the reactive oxygen compounds, and still an oxidant but is less reactive than ozone itself.

Membrane-associated “ozone peroxides” could act as second messengers via cysteine residues and/or reduction through glutathione GSH in a less aggressive way than the superoxide radicals ·O-O^−^ and H_2_O_2_, and take over the regulation of the antioxidants, i.e., without SOD (super oxide dismutase) and CAT (catalase) demand as in the oxidative stress processes of relevant pathological conditions.

The short-chain “ozone peroxide” with its low tendency to radical reactions could initiate the regulation of antioxidant protective mechanisms as redox signal, e.g., via the nuclear factors NFkB and Nrf2 in stress and inflammation processes. Ozone itself is known to upregulate NFkB under special conditions, whereas cortisone blocks this nuclear factor [14].

H_2_O_2_ formed from ozone peroxides, as proposed by other authors, e.g., [10], will probably not fulfil this function, as the H_2_O_2_ content is as a rule pathologically increased in the patients concerned. The corresponding signal transduction is insufficient with a deficit in antioxidant enzymes as a consequence.

#### 3.1.3. Signal Transduction and Bioregulation. The Role of Glutathione

**Figure 7:**
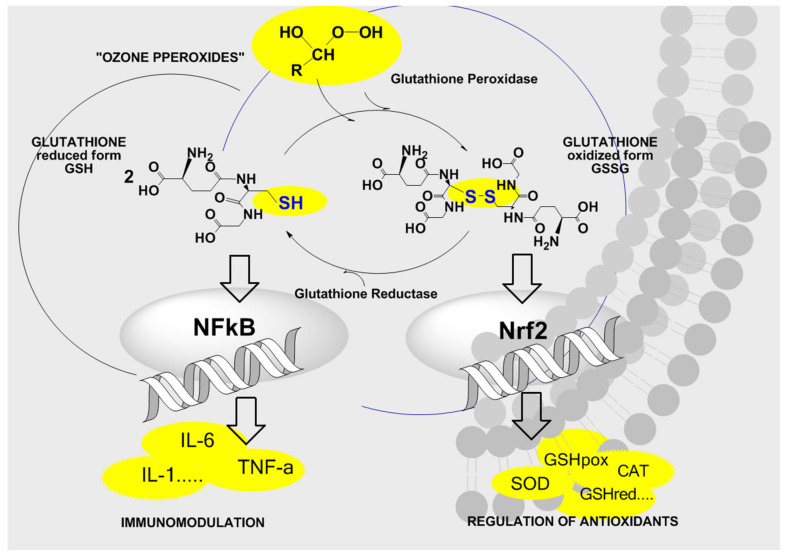
Pharmacological effects of ozone: signal transduction via glutathione and “ozone peroxides”. An intact GSH/GSSG balance is the limiting factor for the ozone concentration and the basis for the low-dose ozone concep revision from [23].

Figure 7 gives a schematic survey of the bioregulation of antioxidants and cytokines through “ozone peroxide” which is directly reduced by the active form of glutathione GSH, the key reaction inducing the regulation. In healthy cells and organs, the reaction H_2_O_2_ + GSH performs the task of signal transduction to the nucleus via the nuclear factors, finally activating or inactivating special DNA segments for the production of the cell specific proteins.

In chronic oxidative stress diseases this information and regulation is completely disturbed in spite of an extremely high content of H_2_O_2_ and the “ozone peroxide” adopts this function, see Figure 1a,b.

The central oxidation of GSH to GSSG (reduced glutathione to the oxidized form in a healthy system 90:10%) in Figure 7 emerges as the ozone concentration and “ozone peroxide” dose limiting factor: the GSH/GSSG balance must not be disturbed in any case, i.e., the reduced form GSH should never drop during a systemic ozone treatment series.

**Figure 8:**
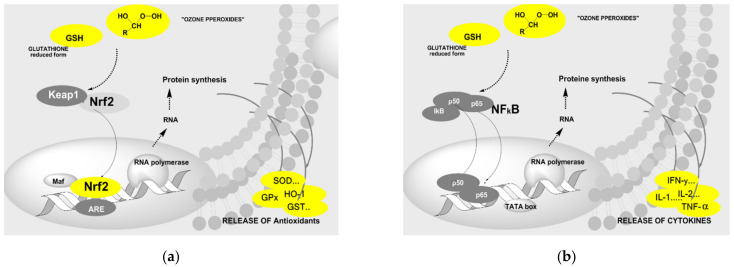
(**a**) The low-dose ozone concept and the regulation of antioxidants, started through the reduction of “ozone-peroxides” by the active (reduced) form of glutathione GSH, initating the signal transduction via the Keap 1/Nrf2–complex and translocation of Nrf2 into the nucleus. In close connection with proteins such as Maf and the ARE complex (antioxidant response elements) the transcription of genes, translation and finally the antioxidant synthesis proceed, see also [15,17,19]. (**b**). Schematic survey of cytokine induction through ozone in mononuclear cells. In healthy organisms, H_2_O_2_ acts as a messenger substance in chronic inflammation where the regulation is out of balance. It is most likely to be “ozone peroxide” replacing H_2_O_2_ and restarting the regulation acc. to [14,19].

The regulation of antioxidative enzymes by ozone, e.g., glutathione peroxidase and reductase, necessary to maintain or restore the glutathione balance, is explained in Figure 8a: Signal transduction is started via the reduction of “ozone peroxides” by GSH inducing the translocation of Nrf2 into the nucleus, transcription, translation, and finally the protein synthesis—in this case antioxidants—proceeds, first proposed by Sagai and Bocci [15,16].

In the meantime, this has been confirmed in different experimental and clinical trials [17,18] and quite recently specified by Togi and collaborators to suggest that initially NFkB is activated and Nrf2 then in a later phase as shown by HeLa cell experiments [19].

In healthy and premalignant tissue, the activation of Nrf2 regulates the cell protection, maintains the cellular homeostasis, and prevents cancer initiation and progression. The contrary can occur in cancer cells whereby a high cellular antioxidant status protects the cell from ROS leading to an increased cancer cell survival and a resistance to chemotherapy [20]. In dependence on the ozone concentrations, this effect has been explored by Costanzo and colleagues with the result that there is no effect on the motility and proliferation of cancer cells in vitro in the systemically used concentrations following the low-dose ozone concept [21,22].

The modulation of the immune response can be understood by a similar mechanism via the nuclear factor NfkB as schematically presented in Figure 8b.

#### 3.1.4. Ozone Specific Stress Markers

As shown above, classic pharmacokinetics with single steps of resorption, liberation, biotransformation and elimination from the body is inappropriate for medical ozone as it reacts instantly with unsaturated fatty acids (single double bond) to “ozone peroxides”, and these, being directly reduced by glutathione GSH, induce bioregulation. These effects are small and measurable, with not less than 3, and usually after 6–10 systemic treatments.

Beside the indication-relevant biochemical and clinical parameters, we measure stress markers, such as antioxidants and cytokines, as a specific response of the body to systemically administered ozone, see Table 3. Preclinical and clinical trials are discussed under this aspect.

## 4. Material and Methods

### 4.1. Rheumatoid Arthritis RA as a Model for Chronic Inflammatory Diseases

In the early stages of ozone therapy, at a time when the effects of systemically administered ozone were not yet fully understood, patients with rheumatoid arthritis received complementary autohemotherapy with ozone at a high dose according to Wolff, H. 1979 [24].

Wrongly, as it turned out, a dose of 6000–10,000 µg ozone in the concentration range between 60 and 80 µg/mL was considered to have an immunosuppressive effect. However, in this case, we are in a toxic range with a high rate of hemolysis. The clinical results were correspondingly useless, with the result that RA was no longer considered to be an indication for ozone.

In the meantime, armed with the pharmacological knowledge that ozone is highly effective as an anti-inflammatory agent in the low-dose range, rheumatoid arthritis has become an exemplary indication for major autohemotherapy and rectal insufflation.

With an ideal concentration range of 20–30, maximum 40 µg/mL and at a dose of 1000–1500 µg, maximum 2000 µg per MAH treatment, or rectal insufflation with a volume of 150–300 mL, respectively are recommended.

Principally, ozone therapy is now considered to be a complementary therapy in RA, enhancing a conventional therapy with the aim of reducing basic therapeutics or making them more tolerable through the activation of detoxifying mechanisms in the liver [25,26,27,28].

### 4.2. Preclinical Trials

**Figure 9:**
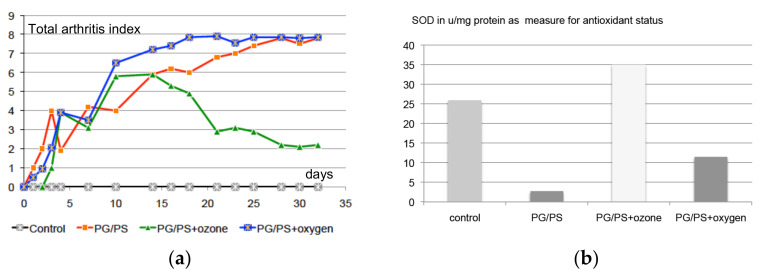

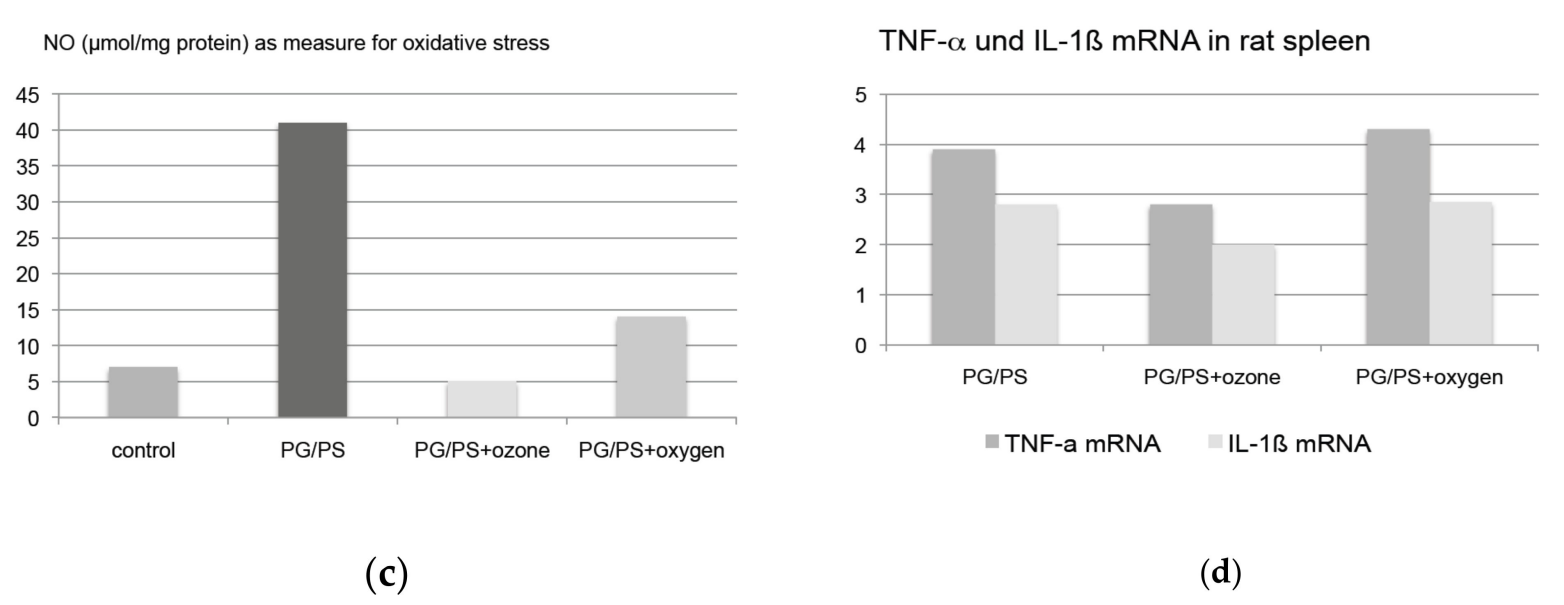
(**a**) Development of the total arthritis index (joint swelling) over 35 days in a preclinical study (*n* = 20) on rheumatoid arthritis RA in rats under intraarticular ozone treatment 3× per week, 20 µg/mL, 0.2 mL, starting on day 10. Control (*n* = 5): intraarticular needle stress 3× per week. PG/PS-induced rheumatoid arthritis (*n* = 15) (peptidoglycan/polysaccharide). PG/PS + ozone (*n* = 5): ozone treatment starting on day 10. PG/PS + oxygen (*n* = 5): oxygen (0.2 mL) treatment starting at day 10 [25]. (**b**) The antioxidant capacity, here as superoxide dismutase SOD in the spleen of RA-rats after 10 intraarticular ozone treatments compared to oxygen treatment and control as described in Figure 9a [25]. (**c**) Oxidative stress, measured as NO in the rat spleen following 10 intraarticular ozone treatments compared to oxygen and control as described in Figure 9a [25]. (**d**) Downregulation of inflammatory stress parameters TNF-α mRNA and IL-1β mRNA, measured in arbitrary units in the rat spleen following 10 intraarticular ozone treatments compared to oxygen as described in Figure 9a [25].

In animal experiments, a considerable improvement in clinically relevant parameters was obtained with medical ozone. This can be seen in Figure 9a using an arthritis index as example: under ozone application, the arthritis index improves systematically, starting on day 10, whereas the administration of oxygen produces practically no change compared with the control: the arthritis index rises continuously and stays at a high level.

As a diradical, oxygen shows a reaction behavior completely different to that from ozone, and tends more to producing a deterioration of the symptoms as expressed, among other factors, by an increase in the inflammation parameters TNF-α mRNA and IL-1ß mRNA (Figure 9d).

**Figure 10:**
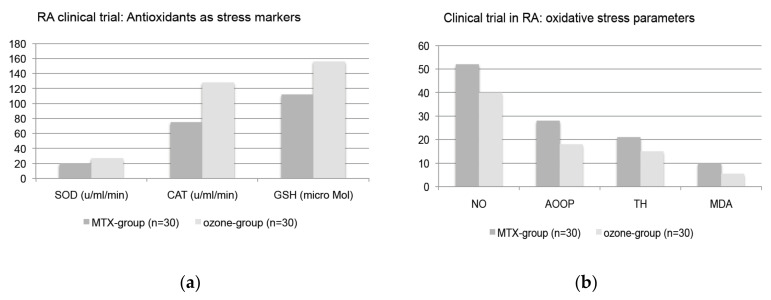

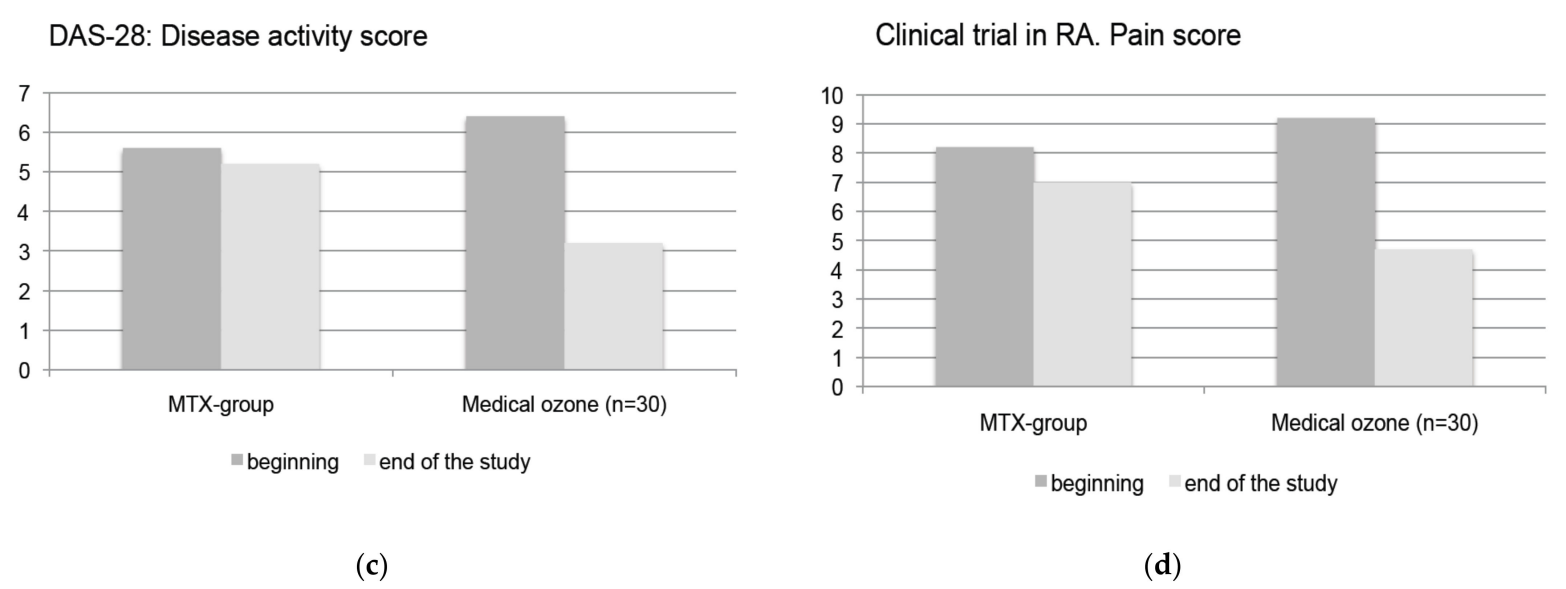
(**a**) Clinical trial in Rheumatoid arthritis RA (*n* = 60) following 20 rectal ozone insufflations with ozone conc.: 25–40 µg/mL and 5000–8000 µg per treatment during 4 weeks. MTX-group (*n* = 30): basic therapy MTX+Ibuprophen+Folic acid. Ozone-group (*n* = 30): basics as MTX-group plus ozone rectally. SOD: superoxide dismutase, CAT: catalase, GSH: reduced glutathione [26]. (**b**). Oxidative stress markers NO: nitrogen monoxide (µMol), AOOP: advanced oxidated proteins (µMol), TH: total hydroperoxide (µMol), MDA: malondialdehyde (µMol) [26]. (**c**). Disease activity score following the EUROPEAN LEAGE AGAINST RHEUMATISM in patients with rheumatoid arthritis treated with standard therapy (MTX-group, *n* = 30) and standard therapy plus rectal ozone insufflation (*n* = 30). DAS-28: low activity y ≤ 3.2; moderate activity 3.2 < y ≤ 5.1; high activity y ≤ 5.1 [26]. (**d**). Pain score in 60 RA-patients. MTX-group (*n* = 30) with basic treatment (see Figure 10a) and ozone group (*n* = 30) with standard therapy plus ozone at the beginning and at the end of the trial after 20 rectal insufflations [26].

Both the preclinical study and the clinical trial show the ozone effect as upregulation of antioxidants and downregulation of oxidative stress as well as the crucial cytokines IL-1, Il-6, and TNF-α. See Figure 9a–d for the preclinical study and Figure 10a–d for the clinical trial.

The regulation of the mRNA (IL-1 and TNF-α) again strengthens the mechanisms of action (low-dose ozone concept) on which the “Guidelines of the Use of Medical Ozone” are based: being a powerful oxidant itself, ozone interrupts the vicious circle of the inflammatory process via the formation of “ozone peroxides”, reduction by cysteine residues and/or glutathione (GSH), bypassing SOD and CAT consumption, then signal transduction and regulation through Nrf2 (antioxidants) and NFkB (immunoregulation).

The pathological concentrations of stress-relevant parameters (H_2_O_2_, MDA malondialdehyde, TH total hydroperoxide etc.) decrease significantly, as well as IL-1, IL-6, and TNF-α. As a consequence, the clinical conditions improve, and the basic medication can be reduced [25].

### 4.3. Clinical Trials

The clinical studies confirm the described mechanism of action of low-dose ozone with the special key position of glutathione: The parameters determined in the preclinical studies show the course expected for the clinical studies: Induction of the cellular antioxidant capacity, reduction of the oxidative stress, and in particular the shift of the GSH/GSSG balance towards the active form: GSH.

This becomes particularly important in the liver with an improvement of biotransformation, which is often highly stressed by the classical medication in RA, especially methotrexate (MTX), and the treatment has to be discontinued. The critical parameter here is γ-GT (gamma-glutamyltransferase), which is significantly involved in the GSH/GSSG balance.

The study design is described in more detail in [26], here only the key points: Randomized clinical trial with 60 patients. All of them (*n* = 60) got the basic treatment with the standardized therapy (MTX + Ibuprophen + folic acid), 30 of them only (MTX-group) and 30 patients, the ozone group, received a complementary ozone treatment in the form of rectal insufflation 5 times per week.

With an increment of 5 µg/mL per week, the ozone concentration was scaled up from 25 to 40 µg/mL.

The biochemical and clinical parameters, here as an excerpt, before beginning and at the end of the treatment concept, are displayed in Figure 10a–d.

In close correlation with the biochemical findings, the clinical data show a significant improvement in the disease activity score (DAS) and pain score in the ozone group; see Figure 10c,d.

To determine the liver protection by rectal ozone insufflation in patients with RA, a controlled clinical trial with 100 patients was performed: *n* = 50 as MTX-group (MTX + Ibuprophen + folic acid) and *n* = 50 as ozone group receiving the same medication + rectal ozone insufflations as described above and in detail in [26,27,28].

**Figure 11:**
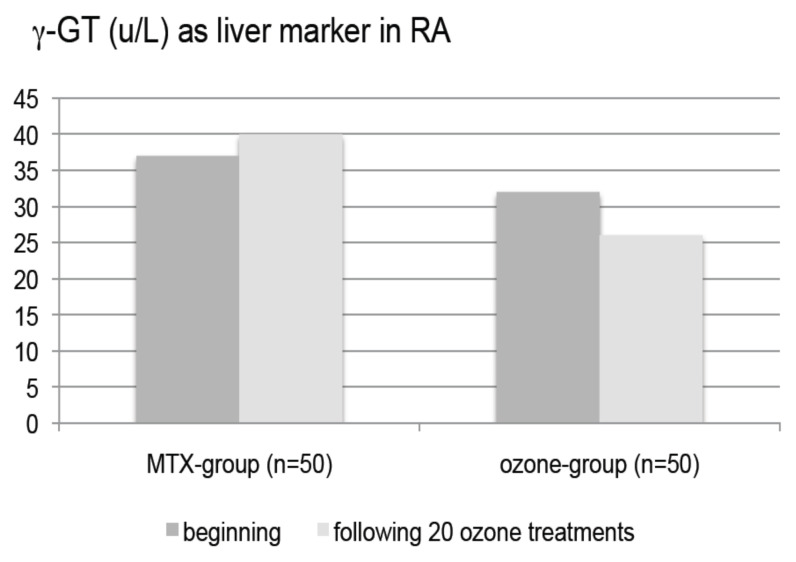
Liver toxicity of standard antirheumatic drugs is reduced by low-dose ozone, here shown as γ-GT (γ-glutamyl transferase) in a clinical trial with 100 Rheumatoid arthritis patients. MTX-group (*n* = 50): Metotrexate + Ibuprophen + Folic acid. Ozone-group (*n* = 50): MTX group + ozone. 20 rectal ozone insufflations 200 mL, concentrations 25–40 µg/mL during 4 weeks [27].

Compared to the MTX-group and as shown in Figure 11, γ-GT decreases in the ozone group in a statistically significant manner [27].

### 4.4. Treatment Concept

In the treatment of rheumatoid arthritis, systemic ozone application has proven itself to be particularly effective, but only in the low-dose range: a combination with standard therapy produces considerably better clinical and biochemical results than standard therapy by itself, and liver-protective mechanisms simultaneously reduce the liver toxicity of basic therapeutics.

Treatment recommendation: Major autohemotherapy MAH or rectal insufflation RI as complement to standard or basic therapy; see Table 4.

## 5. Conclusions

The low-dose ozone concept with its moderate oxidative stress represents an ideal hormesis strategy. Dose–response and concentration–effect relationships in the context of specific applications allow one to fix concentration ranges with therapeutical benefit. Based on the well-known reaction mechanisms of ozone, with its biochemical and pharmacological effects partly shown here, international guidelines have been defined concerning physiological and ozone resistant materials, indications, applications, and the effective concentration and dosage range depending on specific indications. Major autohemotherapy and rectal insufflation have proven themselves as evidence-based systemic forms of ozone application [23].

The pharmacological background investigated in a remarkable number of cell experiments, preclinical and clinical trials is well documented and published in internationally peer reviewed journals. This should encourage clinicians to set up clinical trials that assess chronic inflammatory diseases, integrating medical ozone as a complement in order to benefit from the bioregulatory mechanisms to achieve synergistic effects and reduce side effects of basic therapeutics and drugs, thus helping to protect the liver and kidneys in particular.

## Figures and Tables

**Table 1:** Indications of the systemic ozone treatments MAH and RI and the underlying mechanisms of action acc. to [9].

Applications	Indications	Mechanims of Action
**Systemic applications**		
1. Major autohemotherapy MAH as an extracorporeal blood treatment and iv reinfusion of the patients own blood via pressure-free drip infusion 2. Rectal insufflation RI	Arterial circulatory disorders Chronic inflammatory processes - Angiopathia, diabetic in particular - Chronic inflammation in orthopedics and rheumatology - Rheumatoid arthritis RA - Chronic intestinal diseases - Chronic forms of hepatitis B and C - Complementary oncology - Age-related diseases	- Activation of RBC metabolism, increase of 2,3-DPG, of ATP, improvement of oxygen release - Activation of immunocompetent cells with regulation of cytokine production, such as interferons and interleukines. - Downregulation of oxidative stress - Regulation of the anti-oxidative capacity by signal transduction
**Contraindications**	- Glucose-6-phosphate dehydrogenase deficiency in RBC (favism, acute hemolytic anemia) - Hyperthyroidism if not controlled - The first 3 months of pregnancy - MAH is not indicated in leukemia	

**Table 2:** Standard procedure of major autohemotherapy MAH [9].

MaH Standard Procedure: 50 mL of Blood + 50 mL of O_2_/O_3_ (or 100 mL of O_2_/O_3_ per 100 mL of Blood)			
Ozone concentration per mL of gas	10–20 µg/mL gas	30 µg/mL gas	maximum 40 µg/mL gas
Ozone concentration per mL of blood = biologically relevant concentration	10–20 µg/mL blood	30 µg/mL blood	40 µg/mL blood
Total ozone amount per 50 (100) mL blood	500–1000 µg per treatment	1500 µg per treatment	2000 µg per treatment

Rectal insufflation. RI is a valid alternative to MAH, particularly applied in elderly patients in whom MAH cannot be performed due to unfavorable vein conditions or in chronic conditions with long treatment periods.

**Table 3:** Ozone-specific stress markers during systemic ozone treatment, recommended to follow the treatment success.

Oxidative Stress	Antioxidant Status	Cytokines
TH total hydroperoxides MDA malondialdehyde NO nitrogen oxide γ-GT γ-glutamyl transferase	total SOD superoxide dismutase	interleukine IL-1, IL-6,
	GSH reduced glutathione	TNFα tumor necrosis factor-α

**Table 4:** Rheumatoid arthritis, treatment recommendation.

Major Auto Hemotherapy	Ozone Concentration	Ozone Volume	Ozone Amount	Treatment Frequency	Number of Treatments
Rheumatoid arthritis					
acute stage	30–35 µg/mL	50 mL (100 mL)	1500–1750 µg (3000–3500 µg per 100 mL of blood)	daily	as per control
non acute stage	20–25 µg/mL	50 mL	1000–1250 µg	1× per week, then every 2nd week	in compliance with the patient
Rectal Insufflation	25–30 µg/mL	150–300 mL	3750–9000	Daily in the beginning, then 2× or 1× per week	in compliance with the patient
